# Surrogacy and “Procreative Tourism”. What Does the Future Hold from the Ethical and Legal Perspectives?

**DOI:** 10.3390/medicina57010047

**Published:** 2021-01-08

**Authors:** Valeria Piersanti, Francesca Consalvo, Fabrizio Signore, Alessandro Del Rio, Simona Zaami

**Affiliations:** 1 Department of Anatomical, Histological, Forensic, and Orthopedic Sciences, Sapienza University, 00198 Rome, Italy; valeria.piersanti@uniroma1.it (V.P.); francesca.consalvo@uniroma1.it (F.C.); simona.zaami@uniroma1.it (S.Z.); 2 Obstetrics and Gynecology Department, Sant’Eugenio Hospital, 00144 Rome, Italy; fabrizio.signore@aslroma2.it

**Keywords:** medically assisted procreation, surrogacy, surrogate motherhood, bioethics, legislation

## Abstract

*Background and objectives*: To explore the ethical and legal complexities arising from the controversial issue of surrogacy, particularly in terms of how they affect fundamental rights of children and parents. Surrogacy is a form of medically-assisted procreation (MAP) in which a woman “lends” her uterus to carry out a pregnancy on behalf of a third party. There are pathological conditions, such as uterine agenesis or hysterectomy outcomes, that may prevent prospective mothers from becoming pregnant or carry a pregnancy to term; such patients may consider finding a surrogate mother. Many issues relating to surrogacy remain unresolved, with significant disagreements and controversy within the scientific community and public opinion. There are several factors called into play and multiple parties and stakeholders whose objectives and interests need to somehow be reconciled. First and foremost, the authors contend, it is essential to prioritize and uphold the rights of children born through surrogacy and heterologous MAP. *Materials and methods*: To draw a parallel between Italy and the rest of the world, the legislation in force in twelve European countries was analyzed, eleven of which are part of the European Union (France, Germany, Italy, Spain, Greece, Netherlands, Belgium, Denmark, Lithuania, Czech Republic and Portugal) and three non-members of the same (United Kingdom, Ukraine and Russia), as well as that of twelve non-European countries considered exemplary (United States, Canada, Australia, India, China, Thailand, Israel, Nigeria and South Africa); in particular, legislative sources and legal databases were drawn upon, in order to draw a comparison with the Italian legislation currently in force and map out the evolution of the Italian case law on the basis of the judgments issued by Italian courts, including the Constitutional and Supreme Courts and the European Court of Human Rights (ECHR); search engines such as PubMed and Google Scholar were also used, by entering the keywords “surrogacy” and “surrogate motherhood”, to find scientific articles concerning assisted reproduction techniques with a close focus on surrogacy. *Results*: SM is a prohibited and sanctioned practice in Italy; on the other hand, it is allowed in other countries of the world, which leads Italian couples, or couples from other countries where it is banned, to often contact foreign centers in order to undertake a MAP pathway which includes surrogacy; in addition, challenges may arise from the legal status of children born through surrogacy abroad: to date, in most countries, there is no specific legislation aimed at regulating their legal registration and parental status. *Conclusion*: With reference to the Italian context, despite the scientific and legal evolution on the subject, a legislative intervention aimed at filling the regulatory gaps in terms of heterologous MAP and surrogacy has not yet come to fruition. Considering the possibility of “fertility tourism”, i.e., traveling to countries where the practice is legal, as indeed already happens in a relatively significant number of cases, the current legislation, although integrated by the legal interpretation, does not appear to be effective in avoiding the phenomenon of procreative tourism. Moreover, to overcome some contradictions currently present between law 40 and law 194, it would be appropriate to outline an organic and exhaustive framework of rules, which should take into account the multiplicity of interests at stake, in keeping with a fair and sustainable balance when regulating such practices.

## 1. Introduction

Medically-assisted procreation (MAP) is a phenomenon deeply rooted in modern society and in constant growth.

There is a wide range of methods available for infertile couples that offer important support for natural fertilization [1,2]. However, according to critics, such techniques, including surrogacy, can result in the commodification of human life and the exploitation of women, which is what makes surrogacy, particularly the commercial kind, so moot from a bioethical standpoint [3].

The phrase surrogate motherhood refers to the process by which a woman, referred to as carrier or surrogate mother, undertakes gestation and delivery upon demand from a commissioning couple or individual, the “intended parents”; this practice uses heterologous fertilization techniques [4]. Surrogacy can be of the traditional or gestational kinds: in the former, the surrogate mother uses her own egg and is artificially inseminated using sperm from the intended father or a donor; gestational surrogacy instead relies on an embryo created through an in vitro fertilization (IVF) procedure and implanted into the surrogate’s womb. The child born from gestational surrogacy has no genetical connection to the surrogate. Commissioning (or intended) parents may be heterosexual or homosexual couples, or even singles.

Surrogacy is not a new solution to the old problem of infertility. Although it has been around for a long time, it has become more common since the American College of Obstetricians and Gynecologists (ACOG) published its first statement on this topic in 1983 [5].

There are those who believe that surrogacy should be allowed because it is advantageous for all parties and prohibiting it would limit the autonomy of sterile couples, and there are those who believe instead that the risks outweigh the benefits.

It should be considered that there are pathological conditions that prevent women from becoming pregnant or bringing a pregnancy to term; in such instances, the only solution could be represented by the location of the uterus. Use of surrogacy may be related to congenital uterine agenesis (Mayer–Rokitansky–Küster–Hauser syndrome), major congenital uterine malformation (hypoplastic uterus and bicornuate/unicorn uterus), outcomes of hysterectomy performed for the most diverse reasons or an acquired condition (intrauterine adhesions and leiomyoma) causing uterine dysfunction with failure of attempts at fertility treatment [6]. In these cases, the use of surrogacy could represent a valid alternative to uterus transplantation, especially in cases of failure of the latter [7,8,9]. Patients with serious medical conditions, such as heart or kidney disease that contraindicate pregnancy, could also benefit from surrogacy; this technique also allows the “social” mother to avoid the risks associated with pregnancy (hypertension in pregnancy, preeclampsia [10], amniotic embolism [11,12], particularly for mothers in old age, infections associated with childbirth [13], etc.). It can also be considered as last resort in cases of recurrent implantation failures in assisted fertilization [14,15].

In any case, many issues relating to surrogacy remain unresolved, with significant disagreements within the medical profession, the medical ethics community, state legislatures, courts and the general public.

When courts are called upon to decide on the issue of surrogacy, they tend to favor several factors, often in conflict with each other: the best interests of the child, the rights of the gestational mother, the genetic link between the child and the genetic parents and the of the objectives of the couple who signed the surrogacy contract. There is no consensus in the legal or ethical communities as to which of these factors should be prioritized [16,17]. The authors sought to shed a light on the legal vacuum that currently exists even in many advanced democracies, which often gives rise to uneven decision-making standards and processes, by exploring the current legislative state of affairs relative to surrogacy. It is argued that such uncertainty risks jeopardizing the rights of children and prospective parents alike. Considering that surrogacy is not specifically regulated in many countries via targeted legislation, the role of courts is undoubtedly essential, particularly to provide safeguards for children born abroad and whose intended parents seek to legally register as their own. In that regard, the authors mapped out the evolution of judicial approaches in Italy, while considering the views and judgments of supranational courts on the issue. Children and families alike do have rights that need to be upheld and risk being prejudiced by the current ambiguity in most countries.

In this sense, it is essential to prioritize the rights of babies born through surrogacy arrangements, whose best interests risk being neglected, for the benefit of greater protection of the rights of the other parties involved [18].

## 2. Materials and Methods

The authors set out to conduct a wide-ranging analysis of legislative frameworks currently in force in major European and non-European countries; the countries herein taken into account were chosen based on their significance in terms of population and economic heft on the global scene. Fifty-eight sources were sifted through, all detailing national pieces of legislation for each country herein analyzed. Only the sources specifically reporting on the regulation of MAP techniques and surrogacy were ultimately drawn upon, with a close focus on the legal status of such techniques, as well as the legal recognition of children born abroad through surrogacy. Whenever available, links to official bills, ministerial releases and recommendations were referenced. Regulations mentioning medically-assisted procreation without any relevance to surrogacy and the legal status thereof were ruled out. Furthermore, as it pertains to European countries, ECHR rulings were referenced, in addition to judgments of the courts of those countries, such as Italy, in which the legislation has made interpretative corrections necessary. Finally, publications in the “PubMed” and Google Scholar databases were taken into consideration, resulting from the inclusion of the keywords “surrogacy” and “surrogate motherhood”, in combination with “legal framework”, “regulations” and “court rulings”, so as to paint as broad a picture as possible of the ethical and scientific debate on assisted reproduction as it relates to surrogacy.

### 2.1. Legal and Regulatory Frameworks in European Countries

Surrogacy is still legal in many other countries in Europe and elsewhere.

In some, the practice is not clearly regulated by existing laws, while, in countries where specific legislation does exist, there is a considerable degree of diversity in terms of judicial approaches.

Indeed, while some states ban surrogacy contracts or make them null and void, others allow such agreements to be enforced. In Italy, the practice of surrogacy, often termed by its detractors “uterus for rental”, is subject to law No. 40 of 19 February 2004: “Regulations on medically assisted procreation” [19].

The legislation currently in place in fourteen European countries has been analyzed, ten of which are part of the European Union (France, Germany, Italy, Spain, Greece, the Netherlands, Belgium, Denmark, Lithuania, Czech Republic and Portugal) and three are non-EU members (United Kingdom, Ukraine and Russia), whose legislative frameworks are summarized in Table 1. 

### 2.2. Surrogacy in Non-European Countries

The legislative frameworks currently in place in twelve extra-European countries was analyzed, and the results are summarized in Table 2. It is noteworthy that large countries such as Argentina, the United States and Japan have no national legislation specifically tailored to surrogacy. In the United States and Australia, surrogacy is regulated at the state and territorial level, respectively, i.e., at the second level of government (in most US states, commercial surrogacy is legal, with varying degrees of restrictions, the sole exceptions being Louisiana, Nebraska and Michigan, whereas altruistic surrogacy is legal in all Australian states and territories except one). The overall scenario in extra-European countries reflects the prevalence of altruistic surrogacy being legal, rather than the commercial one (the main exception being Russia), not unlike what is observed in European countries. Israel has the unique feature of “state-controlled surrogacy”: each and every contract needs state approval by a government-appointed committee.

## 3. Results

Various rulings by the European Court of Human Rights (ECHR) [85] and the Italian Constitutional Court, as well as individual Italian local courts, have intervened in the matter of heterologous PMA, providing at times conflicting interpretations of the specific legislation [86,87]. Hence, although Italian jurisprudence has declared the illegitimacy of the ban on heterologous MAP practices, surrogacy is still illegal, as is in France, Spain and Germany. In Belgium and the Czech Republic, there is no specific legislation in this regard. One of the European countries to allow this practice is the United Kingdom, which allows altruistic surrogacy only, as does Denmark. The mother cannot receive any compensation other than compensation for the expenses incurred during the pregnancy. The practice is also legal in Russia and the Netherlands.

In Greece, surrogacy is allowed, but, to legally resort to it, permission from a court of law is required, certifying that the person or couple requesting it cannot otherwise have children.

On the other hand, surrogacy is allowed in all non-European countries herein examined, with the exception of Argentina, Brazil, and some US states. India banned commercial surrogacy in 2018, whereas altruistic surrogacy is legal for residents.

Countries that allow commercial surrogacy are Russia, Ukraine, Thailand (with restrictions designed to curb procreative traveling) and some US states.

## 4. Discussion

Infertility is a condition that affects an increasing number of individuals around the world.

The World Health Organization (WHO) has recognized that infertility should be considered a disease in all respects, affecting the health and well-being of the people who suffer from it.

Reproductive problems and infertility have always exerted a strong psychological pressure.

The relevant developments in the field of medical technologies have provided more and more solutions to the problem of infertility and perspectives, such as surrogacy agreements, have opened the way to new frontiers, raising many ethical and legal issues and discussions and blurring the boundaries between the biological and social bases of kinship [88,89].

The availability of new reproductive technologies has led not only to the separation between procreation and sex, but also to the redefinition of the terms mother and family [90]. With the practice of surrogacy, a subdivision of motherhood has also been created, resulting in a genetic mother, a gestational mother and a social mother [91].

First and foremost, it should be emphasized that heterologous MAP aims to favor life, but still poses significant issues as to the circumstances following birth [92]. Indeed, medically-assisted reproductive methods have given rise to profound social and ethical quandaries that affect the very nature of family relationships, as well as the inherent value of the human embryo; the surrogate embryo transfer technique has further complicated the issue of parenting, making it possible to distinguish among genetic mother, gestational mother and intended mother [93].

The legitimacy of heterologous MAP in some countries poses the problem of financial discrimination against couples, denying those without the necessary means the opportunity to avail themselves of heterologous fertilization abroad, i.e., “fertility tourism”, aimed at circumventing the existing ban.

The development and use of technologies for non-coital reproduction, including surrogacy, also raise the ethical problem of the impact on women and children of a market for materials and services, which according to some constitutes an ethically intolerable commodification of life [94]. Critics consider this a noxious economization of the social realm. As a matter of fact, although surrogacy is often deemed to be a “treatment” option for infertile couples or individuals or an alternative to adoption, and therefore celebrated as instrumental in realizing people’s wishes to be parents, it also entails multiple, extremely complex ethical issues revolving around exploitation, inequality, gender, labor and commercialization [95]. This is particularly true with regard to same-sex couples seeking to become parents; in Italy, for instance, although the legislature enacted a law on civil unions which was also meant for gay couples, second-parent adoption was removed from the bill and is still therefore not legally recognized. While unrelated to surrogacy, it shows how contentious and polarizing an issue same-sex parenting unfortunately still is in some countries, which makes it less likely to gain a favorable parliamentary majority, at least in the short to medium term [96]. Similarly, even in countries where surrogacy is legal for heterosexuals, gay couples or singles cannot resort to it in countries such as India, Russia, Nigeria and Thailand. Overall, the prime ethical concerns raised by surrogacy has to do with exploitation, often coupled with coercion; in cases of commercial surrogacy, i.e., when women are paid to act as surrogates, there is a high risk that the choice to partake in the process may not be fully free, especially in cases where substantial wealth and power differentials exist between intended parents and women acting as surrogates.

### 4.1. Commercial Surrogacy and the Risk of Exploitation: Is Banning Really the Answer? Not so Fast

Commercial surrogacy has been termed by many critics with the charged and pejorative phrase “global baby business” [97], and, before it was banned in India, in December 2018, it was estimated to be worth somewhere between $500 million and $2.0 billion [98]. The seemingly rising international trend of procreative traveling can be ascribed to the opportunities created by assisted reproductive technologies (ARTs) such as in vitro fertilization (IVF), in addition to the relative ease to travel in a globalized world. Starting a family is therefore no longer exclusively associated with intercourse, intimacy and the traditional notion of family: the above-mentioned procreative technologies have in fact created a rift between sexuality and procreation [99]. Consequently, surrogacy has become a feasible and even attractive alternative for many couples when infertility or sexual orientation rule out a “natural” pregnancy or when a woman is unwilling to undertake a pregnancy. Nowadays, although surrogacy is banned in many countries, some others allow altruistic surrogacy, whereas, in others (e.g., India and Ukraine), even commercial surrogacy has become a well-established form of fertility tourism. Evidence seems to suggest that such an industry will further develop in the coming years. Various bioethicists have denounced commercial surrogacy as an arrangement at high risk of exposing women to exploitation [100], stressing the precarious socioeconomic background of a significant number of women, especially in developing countries, who may have decided to become surrogates solely to alleviate their poverty; it would be wrong to engage in broad generalizations when dealing with such a complex and multifaceted issue; nonetheless, a risk does exist that at least some women who decide to be surrogates may not have had a fully free choice. As pointed out by several bioethicists and sociologists, because of the financial incentive, the surrogate’s decision may not be autonomous (which is the basis for ethically tenable actions) but rather heteronomous, i.e., induced by external factors and motives. Indeed, the financial gain for women can play an important role, particularly in developing countries, in determining, or at least influencing, their choice to enter into a surrogacy agreement. In some cases that have been documented, woman who become surrogates can guarantee their families’ livelihood for years, and possibly even offer their own children a better future through educational opportunities that would otherwise be foreclosed [101,102]. According to that argument, surrogates from economically disadvantaged countries cannot validly consent because their background circumstances are essentially coercive, making it all but impossible to make a free choice. In addition to the ethically controversial status of financial compensation under surrogacy contracts, other factors need to be taken into account: the educational level of the surrogates in fact plays a key role in terms of enabling them to fully understand the contract conditions, the risks involved, and even the medical procedures which they will have to undergo. Hence, it has been denounced that, in some instances at least, surrogates might make their decision without full awareness and under circumstances far from ideal. This may negatively affect the whole informed consent process, which under such conditions would be hardly acceptable according to international clinical standards [103]. On the other hand, some make the argument that commercial surrogacy is in itself a form of debasement of human dignity and worth [104]; we believe such a conclusion is faulty, since it does not account for the broad and diverse range of values in modern societies. In addition, arguments based on the assumption that the alleged “commodification” of the parties involved will necessarily result in their mistreatment or harm do not seem to be substantiated by conclusive evidence either [105,106]. Conversely, meaningful data from studies centered around surrogates and the dynamics surrounding surrogacy agreements highlight that the altruistic element, i.e., helping couples with fertility issues achieve parenthood, is the driving motivation for most surrogates. It has also been pointed out that, even though most surrogates do not think of themselves as mothers, they frequently seek to keep a degree of contact with intended parents and children, and that is a key factor in terms of both satisfaction and emotional stability [107]. Those findings and perspectives from scholars actually dealing with surrogate mothers, their family members and intended parents through extensive field research seem to refute most of the arguments and concerns voiced by bioethicists that have been herein summarized [108,109,110]. It is also worth stressing that merely banning commercial surrogacy cannot be enough to solve the underlying issues: prohibition can in fact give rise to an underground market, very likely to jeopardize and harm the interests and rights of women in need, who will seek to improve their financial hardships by partaking in illegal and disguised surrogacy [111]. In addition, as pointed out by some observers [112,113], signaling altruistic surrogacy as the only lawful alternative may ultimately amount to a total deregulation of surrogacy, which in turn could lead to even worse exploitation of women. According to that line of reasoning, an effective way to tackle exploitative surrogacy practices would be to enact labor laws aimed at safeguarding women who choose to be surrogates, as valuable and indispensable elements of the workforce. This approach would entail the legalization of commercial surrogacy, along with strictly enforced regulations aimed at ensuring the women’s dignity and integrity is never compromised and their right to choose is upheld at all times throughout the process [114]. Legal counseling along with medical and psychological screening are of utmost importance to that end. Certainly, given the highly controversial nature of such practices, health professionals who decide to invoke conscientious objection should be allowed to opt out, as it happens in most nations where conscience clauses are enforced in regard to other morally contentious practices such as abortion or assistance in dying; a duty to refer patients to other professionals willing to carry out the procedures in a timely fashion should also be instituted [115,116]. Such a well-balanced approach would in our view be effective at preventing, or at least limiting, the reproductive exploitation of those more vulnerable in society.

### 4.2. The Rights of Children Born through Surrogacy Abroad: Uneven Norms and Uncertainty Go against Their Best Interests

As for the children born through surrogacy abroad, reproductive travels do entail the risk that children born abroad could be denied legal registration in the intended parents’ country of origin, particularly when donor gametes have been used (i.e., when the child is not biologically related to both intended parents). This can only be fixed through a legislative intervention designed to uphold the child’s best interest, which we believe coincides with the right to family life as enunciated in the European Convention of Human Rights, Article 8. In fact, to complicate things further, although Italian statutes prohibit heterologous fertilization and surrogacy, there is currently no targeted legislation aimed at regulating the legal registration of children born through these procedures abroad [117], not even in other EU Member States. This could lead to the risk that, if the intended parents’ country of origin does not recognize the child’s legal parentage and citizenship, they may end up orphaned and without citizenship. There is currently no international set of norms aimed at encouraging the harmonization of national laws and upholding the rights and interests of children born through surrogacy, surrogate mothers and intended parents (whether couples or singles, heterosexual or homosexual). Although it is essential to put in comprehensive sets of safeguards for surrogate mothers, just as much effort needs to be put into preserving the well-being and future prospects of the children [118]. It is in fact unacceptable for some of those children to end up stateless for years due to conflicting national statutes and policies [119].

The European Court of Human Rights (ECHR) therefore faces the difficult task of trying to enforce Convention rights in the context of surrogacy. It is argued that, although there are tensions between the main cases, they can be reconciled by prioritizing the right to personal identity, as an integral part of the right to respect for private life. Case law imposes obligations on Member States in terms of evenly defining the legal status of surrogate children in both cross-border and domestic surrogacy. The very notion of the right to individual identity should be ascribed a wide-ranging interpretation in order to encompass the child’s relationship with genetic, gestational and intended parents; therefore, if the child’s best interest is to be guaranteed, a narrow margin of appreciation should be applied to all state interventions concerning the legal status of children born through surrogacy abroad, so as to achieve, at least within the EU, the highest possible degree of legislative harmonization [120,121].

## 5. Conclusions

The authors believe that, to minimize the risks posed by commercial surrogacy, it may not be enough for national legislatures to devise new laws, or amend existing ones, in isolation. Certainly, while improvements of national laws are obviously necessary, what is missing right now is a collective response at the international level. This is because, in our view, trying to right the wrongs through national initiatives (the way India has resolved to do) cannot conclusively fix the underlying issues.

Surrogacy agencies could take advantage of the loopholes that exist in the law. Eggs, sperm, embryos, surrogates and intended parents could simply move to countries where commercial surrogacy is still legal. Finally, let us remind ourselves that, when one industry is banned in a country, another one can promptly and easily open for business elsewhere (possibly favoring underground, unregulated and unsupervised agreements and procedures liable to harm vulnerable women even more). That seems to be the case in Ukraine, which is quickly becoming a commercial surrogacy “promised land”, now that other countries have banned or severely restricted the practice.

From a social standpoint, banning commercial surrogacy altogether would only risk making things worse overall, as Wilkinson cogently posited. In that respect, it is essential to weigh the implications of prohibiting international commercial surrogacy on the various groups involved. Firstly, the surrogates and their families: if their living conditions are as desperate as exploitation critics argue, it is then quite likely that prohibition will considerably damage women who would have otherwise improved their financial conditions through surrogacy. Since criticisms based on exploitation point to the lack of decent alternatives, it could be reasonably assumed that such women will be unlikely to find equally profitable alternative ways to improve their financial hardships. As many researchers herein cited have remarked, governing, rather than banning, a phenomenon that is obviously not going away by itself anytime soon is probably the best way to go: the ultimate goal is in fact to ensure that the dignity, integrity and self-determination of women are upheld at all times, at every stage of the surrogacy process, and the rights and well-being of children are guaranteed afterwards. Secondly, as far as commissioning parents are concerned, prohibiting international commercial surrogacy would likely prevent at least some of them from having their own children. Others, as argued above, would probably decide to enter into unlawful surrogacy arrangements, either within their own country or by crossing borders, which could well favor criminal elements dedicated to such forms of trafficking (police raids in Cambodia have recently exposed a “surrogacy ring”, for which scores have been charged with cross border human trafficking). Besides, as Italy has shown, national legislation on such sensitive and polarizing issues is often bogged down by partisan in-fighting within legislatures. Although sixteen years have passed since the enactment of law No. 40/2004, and despite the scientific and legal evolution on the subject, no legislative intervention has been made to lay out a well-balanced set of regulatory requirements on the subject of heterologous MAP and surrogacy, either for heterosexual or homosexual couples.

In light of the fact that such procedures are legal in several European and non-European countries, and considering the possibility of traveling to such countries to make use of them, as it happens in a relatively significant number of cases, there is growing awareness that the Italian regulatory framework is ineffective in preventing fertility travels. For this reason, and particularly in order to overcome some of the inconsistencies in the existing Italian legislation (e.g., between law 40 and law 194), there is a pressing need to outline an organic and comprehensive regulatory framework by taking into account the manifold interests and rights to be upheld and, ultimately, strike a fair and respectful balance for all parties involved, so as to ward off any ambiguity that might impinge upon the fundamental reproductive rights of all.

The main effort, however, needs to be made at the international level. It is of utmost importance to pursue an international consensus on surrogacy, which can evolve into a joined-up legislative approach. Despite the inevitable difficulties of arriving at a global agreement, i.e., getting people from different places, cultures or backgrounds to espouse a common set of values, concerns about exploitation and acceptable ethical standards—particularly in terms of upholding the rights of the most vulnerable—represent a significant reason to work towards common ground and over time, a global approach to surrogacy arrangements. Surrogacy as a means to further reproductive rights for all is worthy of a global conversation.

## Figures and Tables

**Table 1:** Surrogacy legislative frameworks in major European countries.

Countries	Legal Status	Legislation
France	Banned	Art. 16-7 of the Civil Code created by law No. 94-653 of 29 July 1994—Art. 3 JORF, 30 July 1994; brokering agreements on procreation or gestation on behalf of third parties is a criminal offence (under Art. 227-12) [20,21].
Germany	Banned	Embryonenschutzgesetz (“Law for the protection of the embryo”), enacted on 13 December 1990, according to which genetic, biological and social motherhood are inextricably bound [22].
Italy	Banned	Surrogacy, often termed by its Italian detractors “uterus for rental”, is subject to law No. 40. The law allows the use of MAP in order to favor the solution of reproductive problems deriving from documented sterility or infertility, provided that it is not possible to remove otherwise the impeding causes of procreation and in the manner prescribed by law, which protects the rights of all those involved, including the child. The law bans heterologous MAP techniques (i.e., those using gametes from third-party donors) and states that, in the event of violation of this prohibition, the gamete donor does not acquire any legal parental relationship with the child and cannot claim any rights or be the holder of obligations against him [23,24].
Spain	Banned	Art. 10 within law 14/2006, passed on 26 May (“Sobre técnicas de reproducción humana asistida”), according to which all surrogacy agreements are null and void [25,26].
Greece	Legal for intended parents in a heterosexual partnership or single females. The latter are required to medically prove their inability to have a pregnancy and be no older than 50 at the time of the contract. Surrogates must be tested for medical and mental fitness.	Law 3305/2005 (“Enforcement of Medically Assisted Reproduction”). Law 4272/2014 has repealed the requirement of permanent residence in Greece [27].
Netherlands	Altruistic surrogacy is legal in the Netherlands, whereas commercial surrogacy is banned. However, few hospitals provide related services in the country, with strict rules to get access, which has resulted in many Dutch couples traveling abroad to seek it.	No targeted legislation. Articles 151b and 151c of the Criminal Code make promoting commercial surrogacy illegal [28,29,30].
Belgium	No official law explicitly forbids altruistic surrogacy, although access is hard to gain. Commercial surrogacy is illegal.	No targeted legislation currently in place [31].
Denmark	Legal, as long as it is done in its altruistic variant and no assisted reproduction techniques are used: the surrogate mother must use her own eggs (traditional surrogacy).	The surrogacy process is not regulated by targeted legislation [32,33].
Czech Republic	There is currently no legislation governing surrogacy, which is considered legal in its altruistic form.	No targeted legislation currently in place, but commercial surrogacy is deemed a criminal offense. The practice is reported to be on the rise, though legal experts warn that any contract or agreements in that respect is unenforceable [34,35,36].
Portugal	Illegal due to Constitutional Court decision, which declared law 25/2016 unconstitutional.	The legislature passed law 25/2016 on 20 July 2016, legalizing surrogacy. On 24 April 2018, and, on September 2019, the Constitutional Court of Portugal overrode several provisions of the law enacted by Parliament, suspected to violate constitutional principles and rights [37,38].
United Kingdom	Altruistic surrogacy is legal. Commercial surrogacy is banned.	Surrogacy is recognized under section 30 of the Human Fertilisation and Embryology Act 2008; In May 2016, a ruling by the Family Division of the High Court of Justice for England and Wales determined that single people can apply to be recognized as the legal parents of a child following a surrogacy arrangement. The law was subsequently changed in December 2018 to allow applications from individual, subject to conditions, including genetic relation to the child. Singles as well as homosexuals seeking parenthood may use it as well, provided that they are UK residents. Surrogacy-related commercial arrangements are prohibited by the 1985 Surrogacy Arrangements Act [39,40].
Russia	Full or gestational surrogacy is legal, i.e., the surrogate mother cannot have a genetic tie with the child. Commercial surrogacy is legal as well.	Gestational surrogacy, even commercial, is legal and available to practically all adults seeking parenthood. A set of medical requirements must be met: Müllerian agenesis, uterine cavity synechia, deformity of the uterine cavity or cervix, somatic diseases contraindicating pregnancy or repeated IVF failure despite use of high-quality embryos [41,42,43].
Ukraine	Surrogacy is legal in all its forms.	Surrogacy is officially regulated by Clause 123 of the Family Code of Ukraine and the order of the Ministry of health of Ukraine “On approval of the application of assisted reproductive technologies in Ukraine” from 9 September 2013 No. 787 [44,45,46,47].
Lithuania	Surrogacy is illegal in Lithuania, and any agreement would be unenforceable.	Article 11 of the Law on Medically Assisted Procreation of the Republic of Lithuania (14 September 2016 No. XII-2608) declares all surrogacy agreements null and void. Moreover, the Lithuanian parliament (Seimas) has recently issued a resolution decrying surrogacy and urging the President and the Ministry of Foreign Affairs to propose amendments to international treaties meant to facilitate a surrogacy ban at the national level. It also calls on the Council of Europe to launch an inquiry into whether existing international laws have been fully complied with by EU member states [48].

**Table 2:** Surrogacy outside of Europe.

Countries	Legal Status	Legislation
United States	There is no federal law governing surrogacy across the country; regulations vary at the state level.	Surrogacy is allowed in most states without any specific legislation regulating its use; 14 states (among which Texas and California) have targeted surrogacy legislation in place; 12 states have strict limitations in place (such as deeming contracts void and unenforceable); only 3 states ban surrogacy altogether (Louisiana, Michigan and Nebraska), whereas New York passed a new piece of legislation, set to go into effect on 15 February 2021, allowing for compensated surrogacy arrangements and provides a Pre-Birth Order, which confirms the legal parentage of the intended parents at the moment of the child’s birth [49,50,51,52,53].
Canada	Altruistic surrogacy is legal.	The Assisted Human Reproduction Act of 2004 criminalizes commercial surrogacy. The validity of surrogacy contracts and the process for establishing parenthood of the child is governed by provincial law. Quebec fails to recognize any surrogacy contracts, whereas British Columbia has the most permissive laws governing surrogacy. Provinces also vary in the degree to which they compensate surrogacy expenses, such as IVF procedures. On 20 February 2020, Canadian Senators introduced Bill S-216, designed “to decriminalize, in certain circumstances, the payment of sperm or egg donors and surrogate mothers” [54,55].
Brazil	Commercial surrogacy is forbidden based on article 199(4) of the Brazilian Federal Constitution that interprets it as a form of human organ trafficking. Altruistic surrogacy is allowed, with limitations, based upon medical guidelines bit not on any specific legislation.	The ban on surrogacy hinges on article 199(4) of the Brazilian Federal Constitution [56]. Due to the lack of legislation, the Federal Medical Council created a guideline for altruistic surrogacy that has been in place since 2010, which constitutes the only set of applicable rules in Brazil to differentiate this practice from commercial surrogacy [57].
Argentina	No surrogacy legislation is currently in place despite an unsuccessful attempt by lawmakers to pass a bill in 2017. Surrogacy contracts are regulated by Civil Code provisions.	The Civil Code was updated in 2014 (Ley 26.994) and contains a special chapter dedicated to regulating parentage when a child is born through an assisted reproductive technique. Commissioning parents have to sign a consent document prior to the IVF procedure; when the child is born, the gestational mother is considered the legal mother; she can then waive her maternity rights and give all rights to the intended parents [58]; fertility tourism in the country, favored by flimsy regulations and inadequate oversight, has been denounced [59].
Australia	Altruistic surrogacy is legal in all jurisdictions except the Northern Territory; commercial surrogacy is a criminal offense.	Australia’s surrogacy regulations are administered at a State level. They all share a common premise: only altruistic surrogacy arrangements are legal. Overseas commercial surrogacy is illegal for residents in New South Wales (NSW), Queensland (QLD), and Australian Capital Territory (ACT) [60,61,62,63].
Israel	Legal with state approval.	The Israeli government legalized gestational surrogacy via the “Embryo Carrying Agreements Law, in March 1996 [64]. That piece of legislation has made Israel the first country in the world to introduce a form of “state-controlled surrogacy” in which each and every contract has to gain the direct approval of the state [65]. A state-appointed committee allows for surrogacy arrangements only by Israeli citizens. In February 2020, the Israeli Supreme Court ruled the restriction on same-sex couples from entering surrogacy agreements as discriminatory, thus giving the state one year to change the law.
India	Currently, only resident married heterosexual couples with infertility problems are allowed to access altruistic surrogacy agreements (only compensation of medical expenses is legal). Commercial surrogacy is not legal in India anymore.	The Indian Government introduced a bill to amend the previous surrogacy law in October 2015, aiming to ban foreign citizens from using surrogacy services in the country and to stop the massive flow of “fertility tourists” looking for a surrogate mother. The bill was passed on 21 November 2016 under the name Surrogacy (Regulation) Bill, 2016 [66,67].
People’s Republic of China	Illegal, yet widespread and tolerated.	In 2001, China’s Ministry of Health released the “Administrative Measures on Assisted Human Reproduction Treatments”, banning any form of trade of gametes and embryos and prohibiting doctors from carrying out surrogacy procedures; the ban was reiterated in 2013. However, the flourishing clandestine commercial surrogacy market is tolerated by the authorities and has been estimated to involve somewhere between 400 and 500 agencies in 2012 [68,69,70,71,72].
Thailand	Only opposite-sex married couples as Thailand residents are allowed to have a commercial surrogacy contract arrangement.	On 19 February 2015, the Thai Parliament enacted a bill meant to regulate surrogacy on its territory. The text put in place major restrictions. It came into effect on 30 July 2015. In the past, Thailand was a popular destination for couples seeking surrogate mothers [73,74,75].
Japan	No legislation governs surrogacy use. Guidelines and legal opinions released by professional societies and government agencies, based on societal and cultural deeply rooted elements, have however stigmatized and discouraged such a practice.	The absence of any formal regulation of gestational surrogacy in Japan has resulted in those who decide to use it seeking overseas surrogates; however, that places the children born of such arrangements at high risk of statelessness upon arrival in Japan [76,77,78,79,80,81].
Nigeria	Although no legal framework is in place for regulating surrogacy, the practice is tolerated in some Nigerian regions, although the legitimacy of such acts remains dubious, given the lack of any law or judicial pronouncement on the practice itself.	Numerous agencies offer surrogacy services to couples struggling to conceive a child. However, sources have pointed out that lack of legislation makes parents and surrogate mothers vulnerable, due to inadequate medical screening, insufficient psychological support and caesarean section overuse. Surrogacy contracts are enforceable in Nigerian courts, which usually follow common law precedents, by recognizing the rights of both parties in a surrogacy contract [82,83].
South Africa	Only altruistic surrogacy is allowed, including for singles and same-sex couples; access is only legal for residents, and surrogate mothers may not be financially rewarded other than compensation for pregnancy-related expenses.	Section 19 of the Children’s Act, which regulates surrogacy in South Africa, entered into force on 1 April 2010. The law strongly recommends that at least one of the future parents provide the gametes for the procedure. The surrogate mother has the right to unilaterally terminate the pregnancy after consulting with and inform the commissioning parents; if she decides to do so for non-medical reason, she may be obliged to refund any medical reimbursements she had received [84].

## Data Availability

The data presented in this study are available on request from the corresponding author.
